# Supplementary material for: Transcriptional rewiring of an evolutionarily conserved circadian clock
Source: EMBO J. 2024 Apr 16;43(10):5. doi: 10.1038/s44318-024-00088-3 (PMC11099105; doi:10.1038/s44318-024-00088-3)
Supplement: Supplementary file 1 — Appendix [file 44318_2024_88_MOESM1_ESM.pdf]

Appendix Figures

**Figure name**	**Page**
Appendix Figure S1 - Evaluation of *ccg promoters*.	2
Appendix Figure S2 - The assessed Hybrid Oscillators failed to display rhythmic behavior after a LL to DD transition.	3
Appendix Figure S3 - FRQ levels in the different HOs.	5
Appendix Figure S4 - The period of the semi-synthetic Oscillator HO-10 is dependent on FRQ determinants.	6
Appendix Figure S5 - Turning on the light fixes the phase of the semi-synthetic oscillator HO-10.	8
Appendix Figure S6 - Oscillation profiles under LD12:12 and LD4:20 entrainment conditions *in silico*.	9
Appendix Figure S7 - Model simulations reproduce the behavior of WT and HO-10 clocks.	10

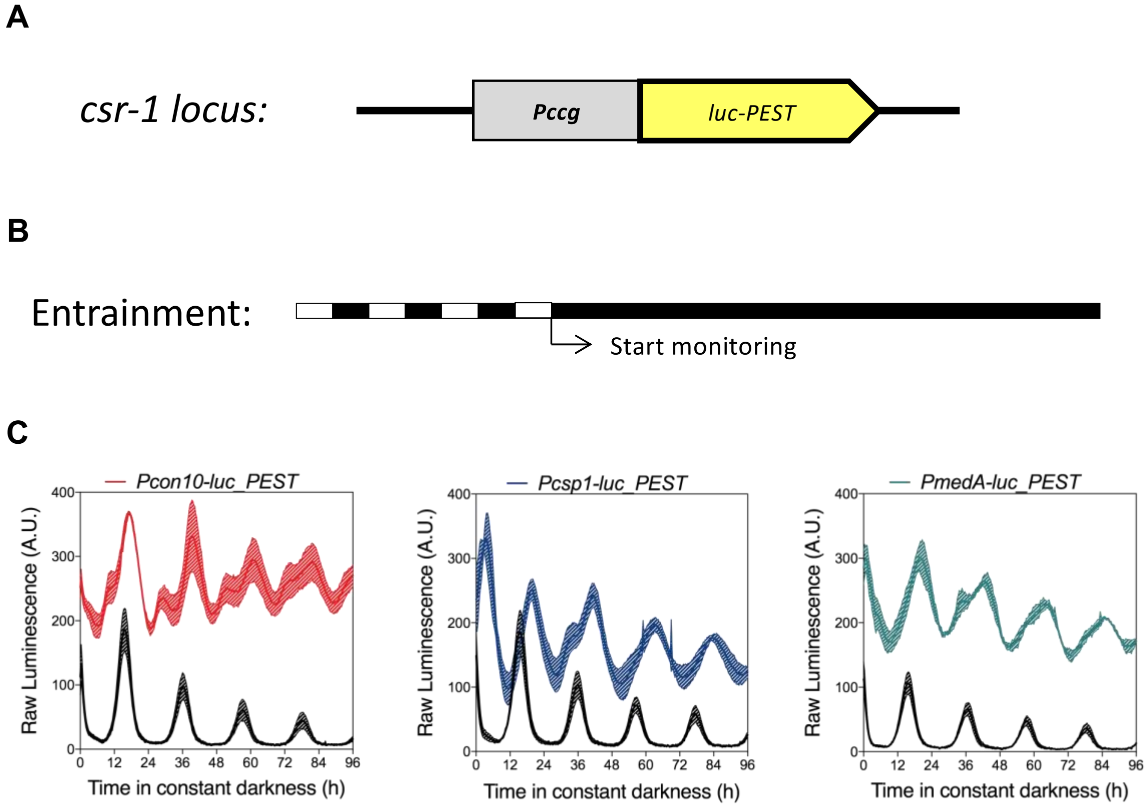

Appendix Figure S1 - Evaluation of *ccg promoters*.

A. Scheme of the constructs utilized for the analysis of *ccg* promoters using *luciferase* as a real-time reporter.

B. Entrainment protocol used to evaluate the different reporters: prior to recording in darkness, the strains were grown for three days under 12:12 LD cycles.

C. Evaluation of different *ccg promoters* under DD conditions. The black traces represent a *frq*$_{c\text{-}box+pLRE}$-*luc_PEST* reporter, while the luminescence of the different promoters is shown in color. In all cases, experiments were run three independent times, and a representative set is shown. Each luciferase trace corresponds to the average of three different wells $\pm$ SD.

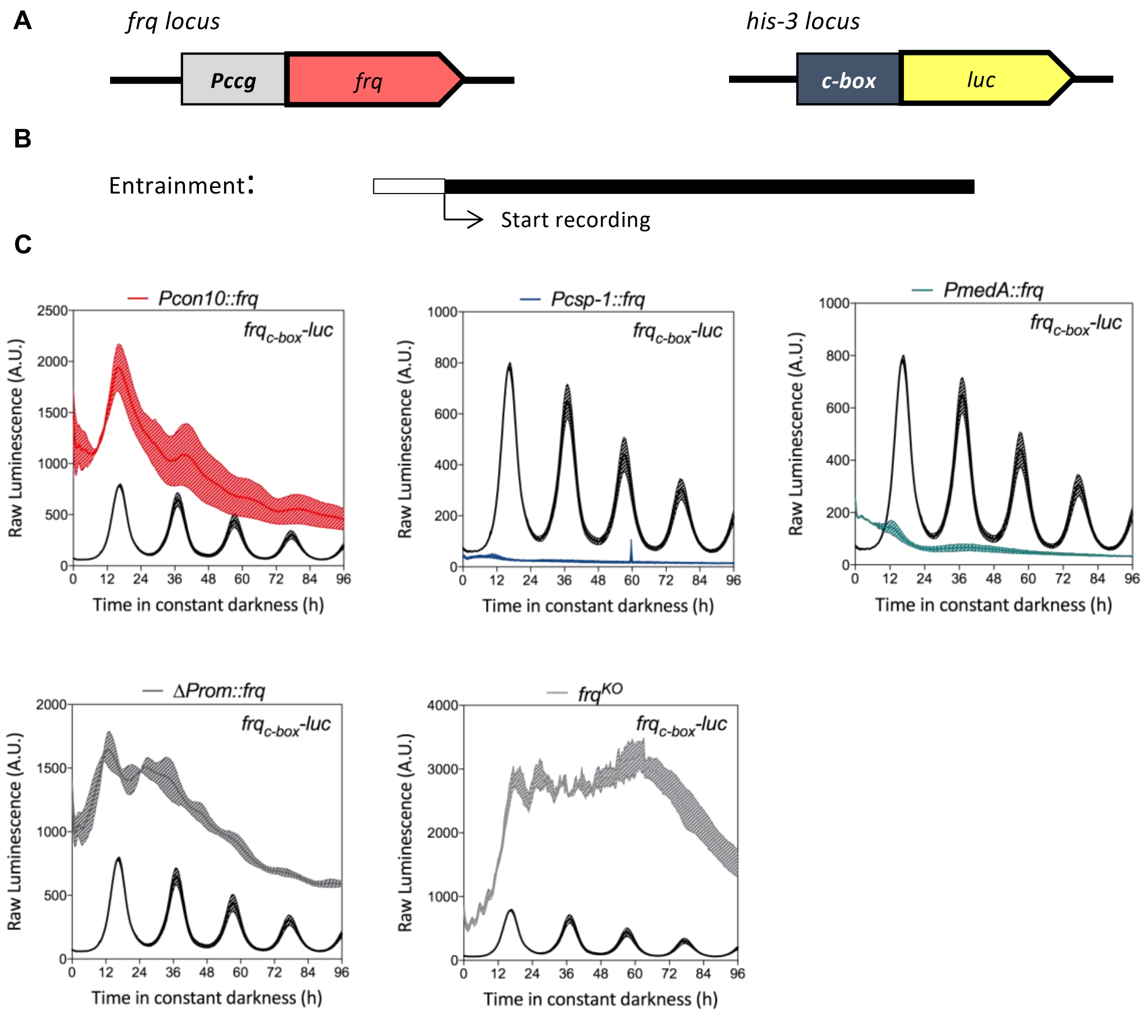

Appendix Figure S2 - The assessed Hybrid Oscillators failed to display rhythmic behavior after a LL to DD transition.

A. The endogenous *frq* promoter was replaced, by homologous recombination, with the indicated *ccg* promoters to generate different Hybrid Oscillators (HO) where *frq* expression is under the control of the specified sequences. Additionally, at a different locus all strains have *frq*$_{c\text{-}box}$-*luc* as a clock reporter which allows monitoring WCC activity.

B. Entrainment protocol used to evaluate the different HO. Prior to recording in darkness, the strains were grown for 24 hours in LL.

C. Evaluation of HOs under DD conditions, by analyzing LUC activity coming from a *c-box-luc* reporter. The black traces represent a WT strain, while the different HOs are shown in color. A negative control without a promoter (Δ*Prom::frq* only containing the drug resistance cassette, *bar*) was examined, showing a behavior comparable to a Δ*frq*. In all cases, experiments were run three independent times, and a representative set is shown. Each luciferase trace corresponds to the average of three different wells ± SD.

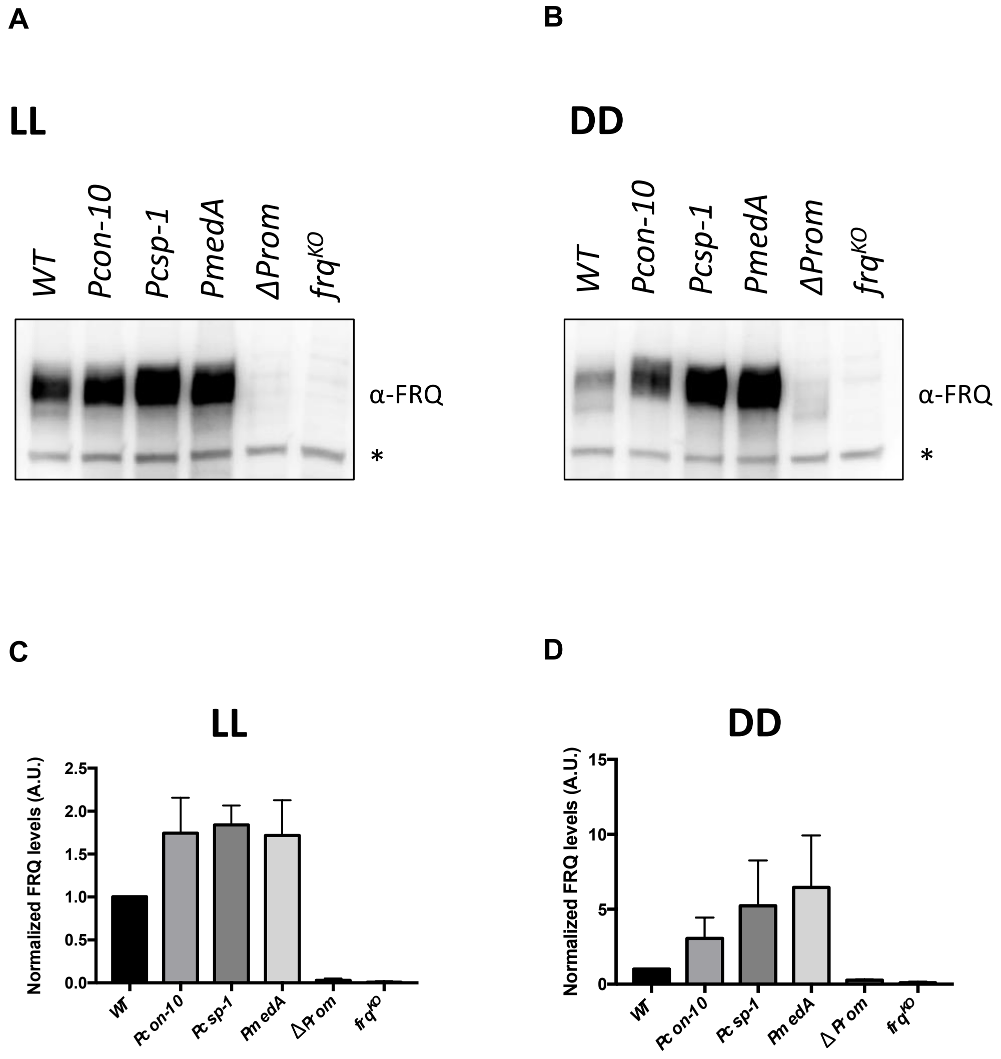

Appendix Figure S3 - FRQ levels in the different HOs.

A, B. Western blots showing the levels of FRQ in the different HOs, after growth in LL for 48 hours (**A**) and after 24 hours in DD (coming from 24 hours in LL) (**B**)**.** The name of each sample indicates the promoter that controls *frq* transcription. * Unspecific band.

C, D. Quantification of the western signals, coming from three replicates with average and SD. Values were normalized with respect to the WT in LL (**C**) and in DD (**D**).

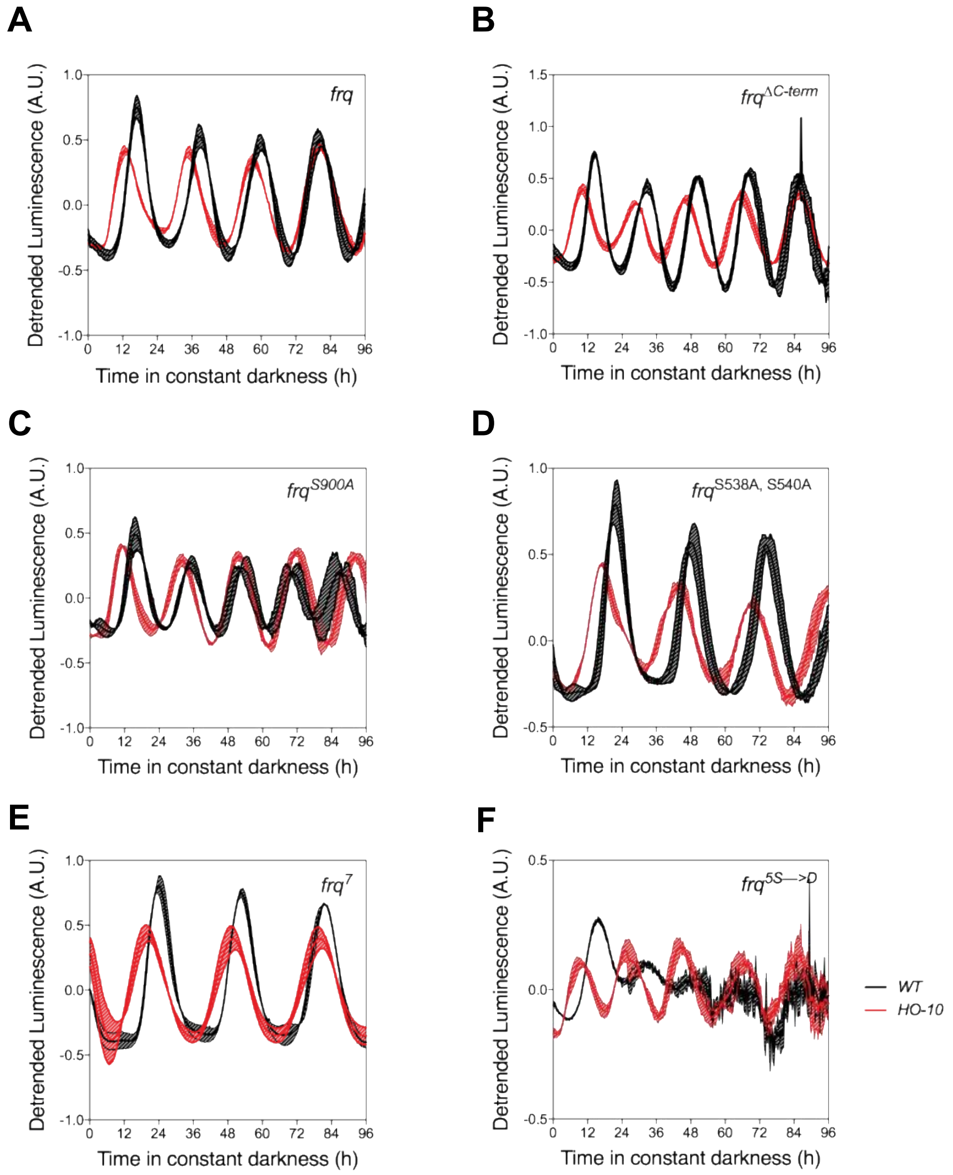

Appendix Figure S4 - The period of the semi-synthetic Oscillator HO-10 is dependent on FRQ determinants.

A-F. The graphs correspond to the normalized detrended curves presented as raw datasets in **Fig. 2**. As indicated therein, different *frq* alleles: *frq* (**A**), *frq*$^{\Delta c\text{-}term}$ (**B**), *frq*^{S900A} (**C**), *frq*^{S538A,S540A} (**D**), *frq*^{7} (**E**) and *frq*$^{5S\rightarrow D}$ (**F**), as depicted in the insets, were analyzed in the context of a WT (black) or the HO-10 semi-synthetic circuitry (red lines), under constant darkness (DD), utilizing *frq*$_{c\text{-}box}$-*luc*

as a reporter. The strains were entrained for three days under 12:12 LD cycles, prior to recording in constant darkness. In all cases, experiments were run three independent times, and a representative set is shown. Each luciferase trace corresponds to the average of three different wells ± SD.

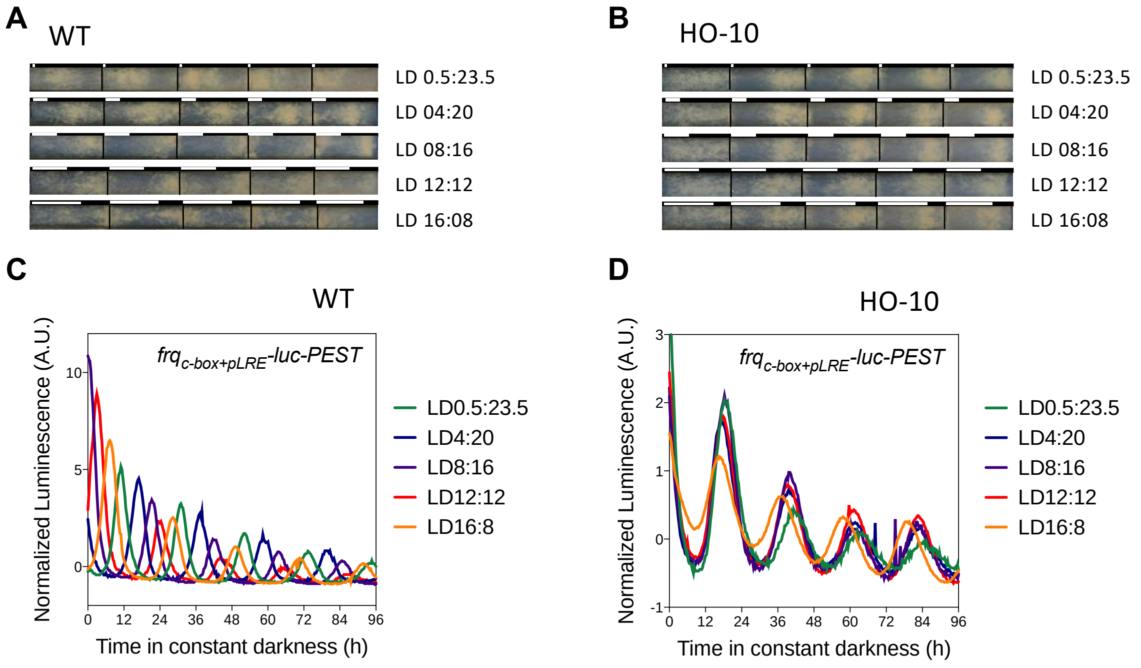

Appendix Figure S5 - Turning on the light fixes the phase of the semi-synthetic oscillator HO-10.

A, B. Race tubes for WT (**A**) and HO-10 (**B**) were grown under the indicated LD regimes, and marked at the moment lights were turned on.

C, D. Luciferase activity was monitored in DD for WT (**C**) and HO-10 **(D),** and recording started at the end of 3 days of the indicated LD entrainment regimes. In all cases, experiments were run three independent times, and a representative set is shown. Each luciferase trace corresponds to the average of three different wells ± SD.

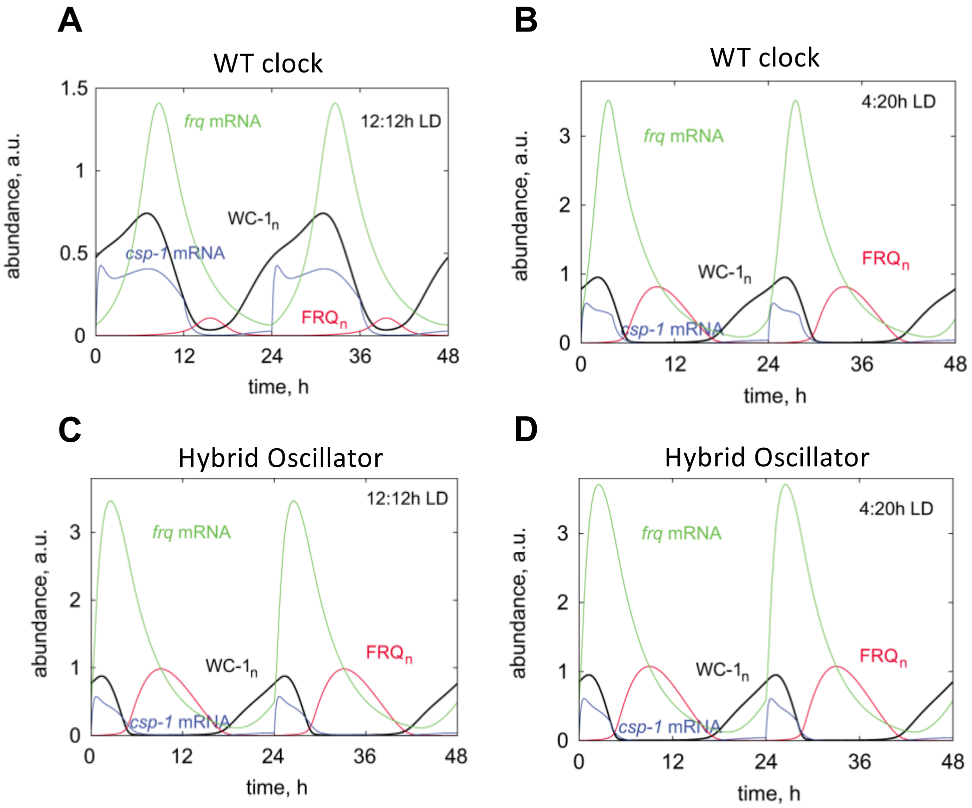

Appendix Figure S6 - Oscillation profiles under LD12:12 and LD4:20 entrainment conditions *in silico*.

A, B. Oscillation profiles in the WT clock in LD12:12 (**A**) and LD4:20 (**B**)

C, D. HO-10 oscillator in LD12:12 (**C**) and LD4:20 (**D**).

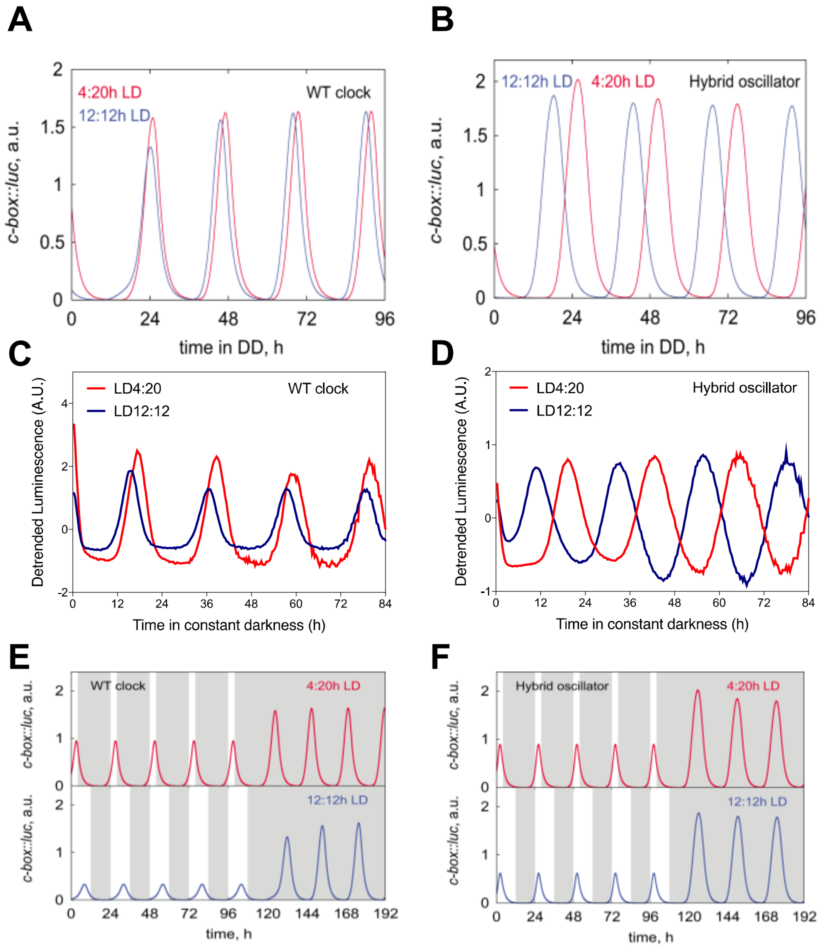

Appendix Figure S7 - Model simulations reproduce the behavior of WT and HO-10 clocks.

A-B. Phase differences in simulated free-running oscillations in DD after LD12:12 and LD4:20 entrainment in WT clock (**A**) and hybrid oscillator (**B**).

C-D. Experimentally luciferase activity observed in DD after three days of LD12:12 and LD4:20 entrainment in WT clock (**C**) and hybrid oscillator (**D**).

E-F. Model oscillations of the WT and HO-10 under two entrainment regimes followed by DD. LD regime is simulated for the first 120 h. Note that free-running oscillations in DD shown in (**A** and **B**) start after the last light phase which corresponds to 100 h for LD4:20 (top panel) and 108 h for LD12:12 (bottom panel) in (**E** and **F**).
