# Supplementary figures and images for: Transcriptional rewiring of an evolutionarily conserved circadian clock
Source: EMBO J. 2024 Apr 16;43(10):5. doi: 10.1038/s44318-024-00088-3 (PMC11099105; doi:10.1038/s44318-024-00088-3)

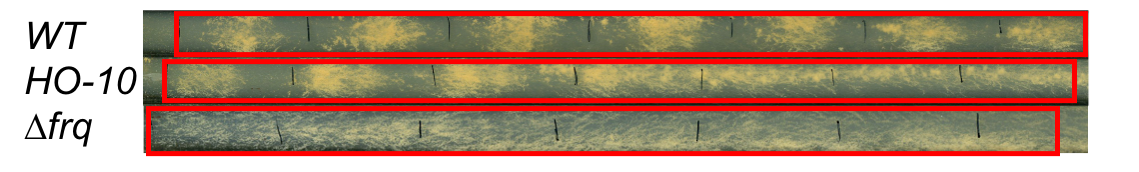

Supplement: Supplementary file 5 — Source data Fig. 1 [file 44318_2024_88_MOESM5_ESM.zip › Source Data Figure 1/Figure 1F/Racetube_Fig1Fnew.tif]

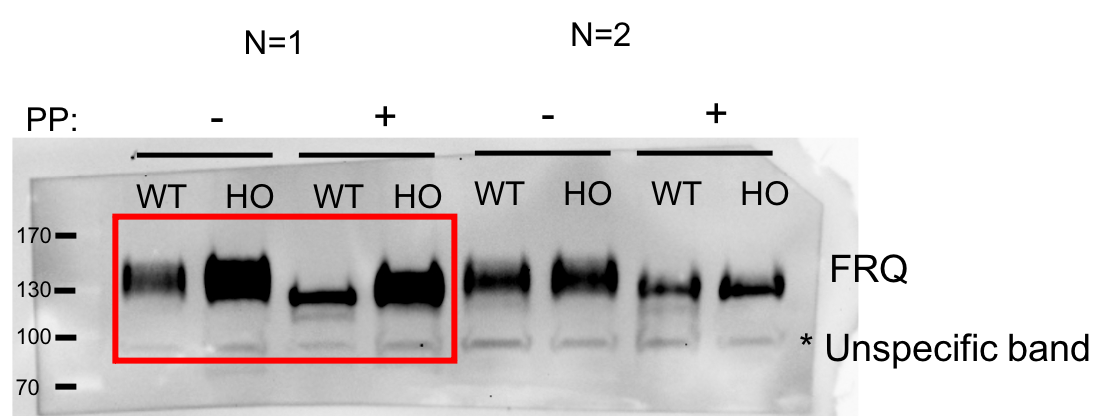

Supplement: Supplementary file 7 — Source data Fig. 3 [file 44318_2024_88_MOESM7_ESM.zip › Figure 3/Figure 3B/western FRQ.tif]

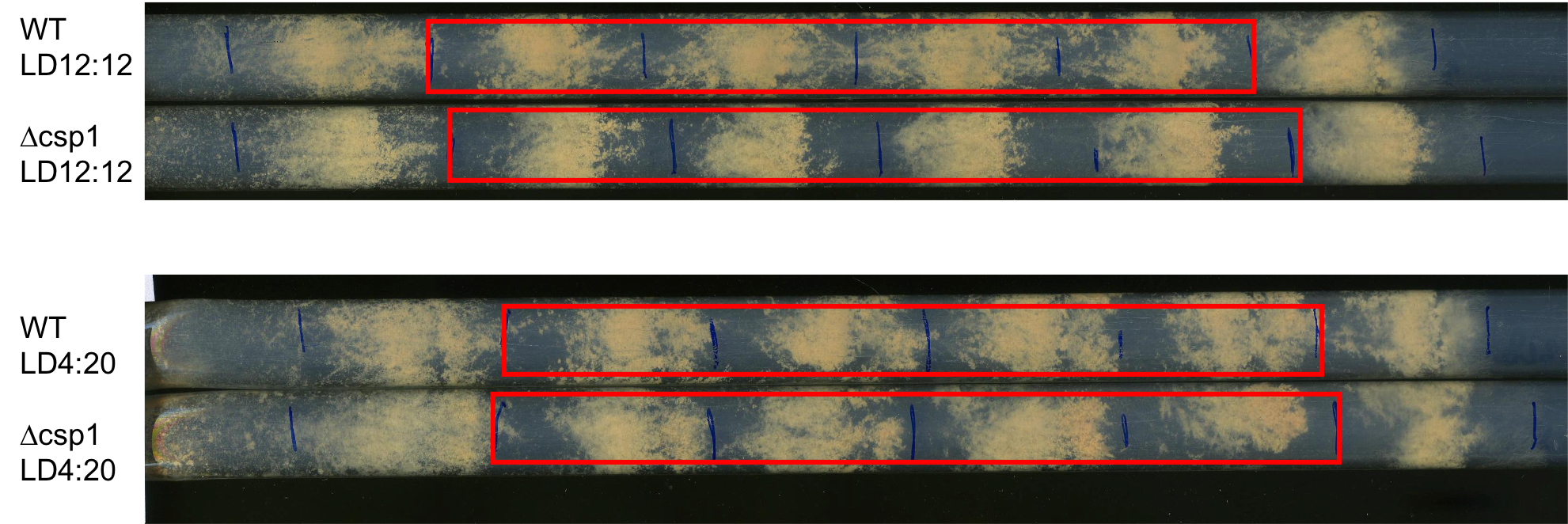

Supplement: Supplementary file 9 — Source data Fig. 5 [file 44318_2024_88_MOESM9_ESM.zip › Figure 5/Figure 5E/Racetubes Fig5E.tif]
