# Supplementary material for: Transcriptional rewiring of an evolutionarily conserved circadian clock
Source: EMBO J. 2024 Apr 16;43(10):5. doi: 10.1038/s44318-024-00088-3 (PMC11099105; doi:10.1038/s44318-024-00088-3)
Supplement: Supplementary file 11 — Expanded View Figures [file 44318_2024_88_MOESM11_ESM.pdf]

Expanded View Figures

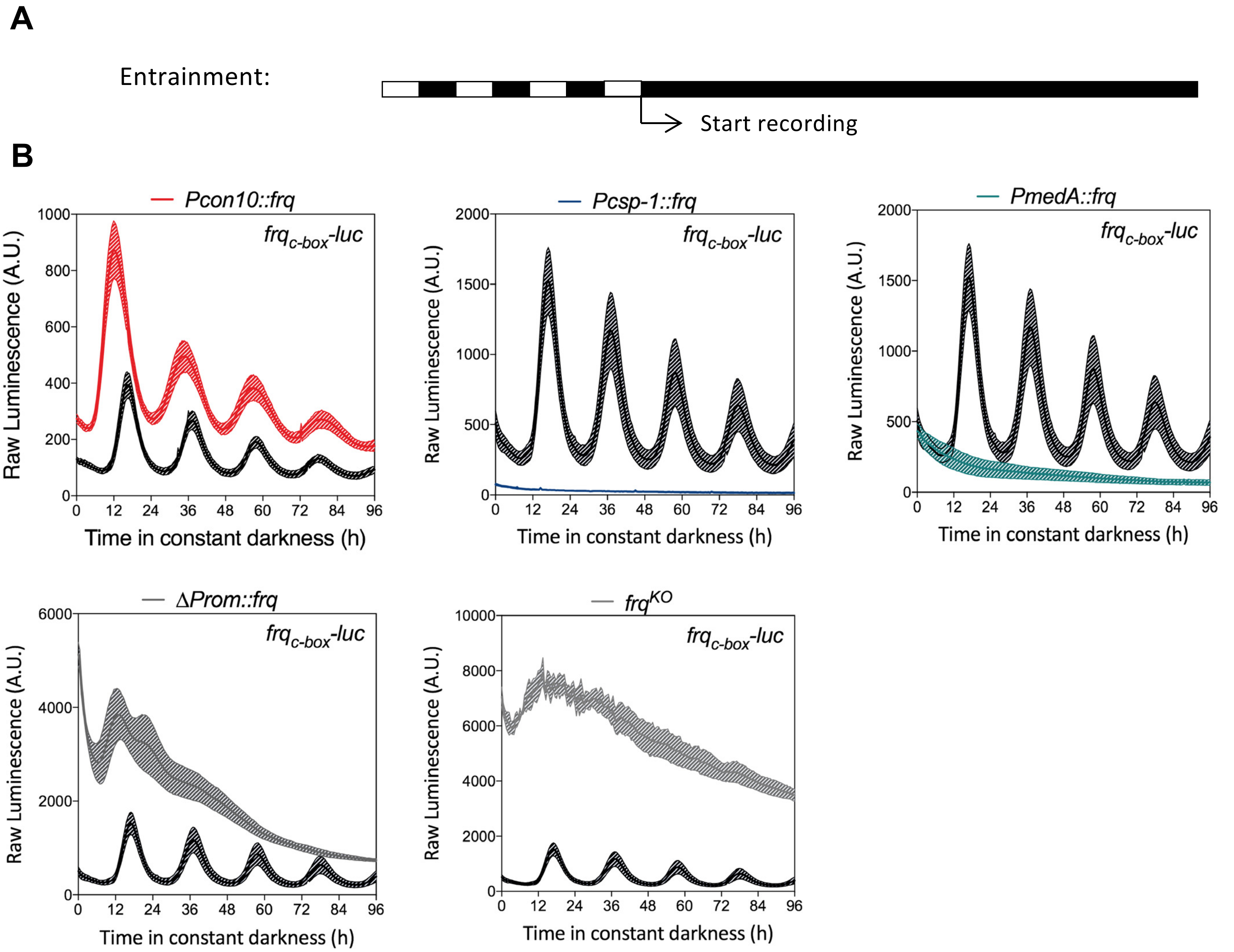

Figure EV1. Only one the tested Hybrid Oscillators sustains robust rhythms after a three days LD 12:12 entrainment.

(**A**) Entrainment protocol used to evaluate the different HOs. Prior to recording in DD, the strains were entrained for three days under 12:12 LD cycles. (**B**) Evaluation of HOs under DD conditions, by analyzing LUC activity coming from a *frq$_{c\text{-}box}$-luc* reporter. The black traces represent a *wild type* strain, whereas the different HOs are depicted in color. A negative control without a promoter (Δ*Prom::frq* only containing the resistance cassette, *bar*), as well as a *frq^{KO}* were examined. In all cases, experiments were run three independent times, and a representative set is shown. Each luciferase trace corresponds to the average of three different wells ± SD.

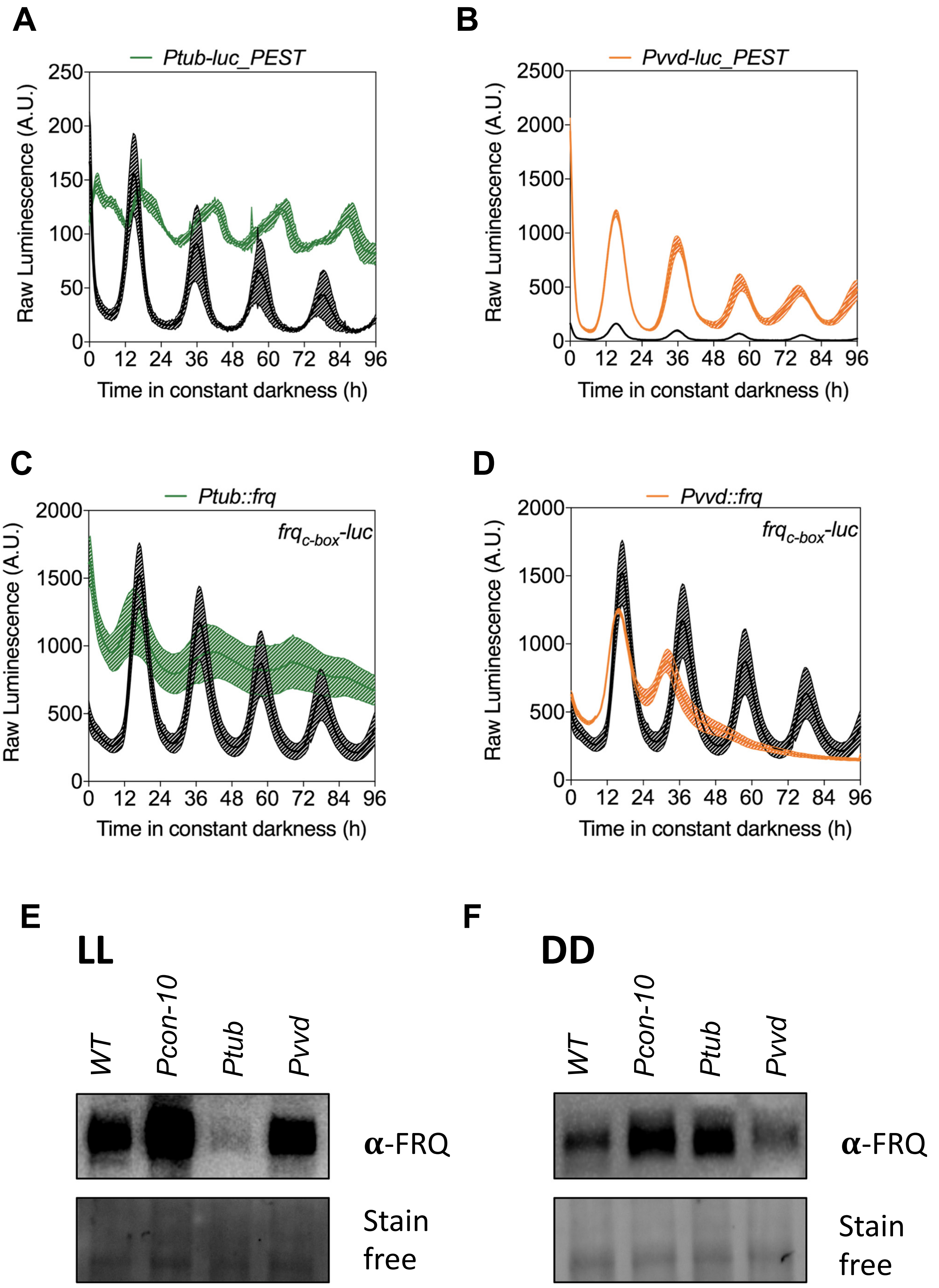
A
Ptub-luc_PEST
Raw Luminescence (A.U.)
Time in constant darkness (h)
B
Pvvd-luc_PEST
Raw Luminescence (A.U.)
Time in constant darkness (h)
C
Ptub::frq
frq$_{c\text{-}box}$-luc
Raw Luminescence (A.U.)
Time in constant darkness (h)
D
Pvvd::frq
frq$_{c\text{-}box}$-luc
Raw Luminescence (A.U.)
Time in constant darkness (h)
E
LL
WT
Pcon-10
Ptub
Pvvd
α-FRQ
Stain free
F
DD
WT
Pcon-10
Ptub
Pvvd
α-FRQ
Stain free

Figure EV2. Hybrid oscillators with *tubulin* and *vivid* promoters exhibit weak rhythms after three days in LD 12:12 entrainment.

(**A**,**B**) Evaluation of *tubulin* (green (**A**)) and *vivid* (orange (**B**)) *promoters* under DD conditions. The strains were grown for three days under 12:12 LD cycles prior start recording in darkness. The black traces represent a $frq_{c\text{-}box+pLRE}$*-luc_PEST* reporter. In all cases, experiments were run three independent times, and a representative set is shown. Each luciferase trace corresponds to the average of three different wells ± SD. (**C**,**D**) Evaluation of HOs under DD conditions, by analyzing LUC activity coming from a $frq_{c\text{-}box}$*-luc* reporter. The black traces represent a *wild type* strain, whereas the different HOs are depicted in color. In green the *tubulin promoter* (**C**) and in orange the *vivid promoter* controlling *frq* transcription (**D**). In all cases, experiments were run three independent times, and a representative set is shown. Each luciferase trace corresponds to the average of three different wells ± SD. (**E**,**F**) Western blots showing the levels of FRQ in the different HOs, after growth in LL for 48 h (**A**) and after 24 h in DD (coming from 24 h in LL) (**B**). The name of each sample indicates the promoter that controls *frq* transcription.

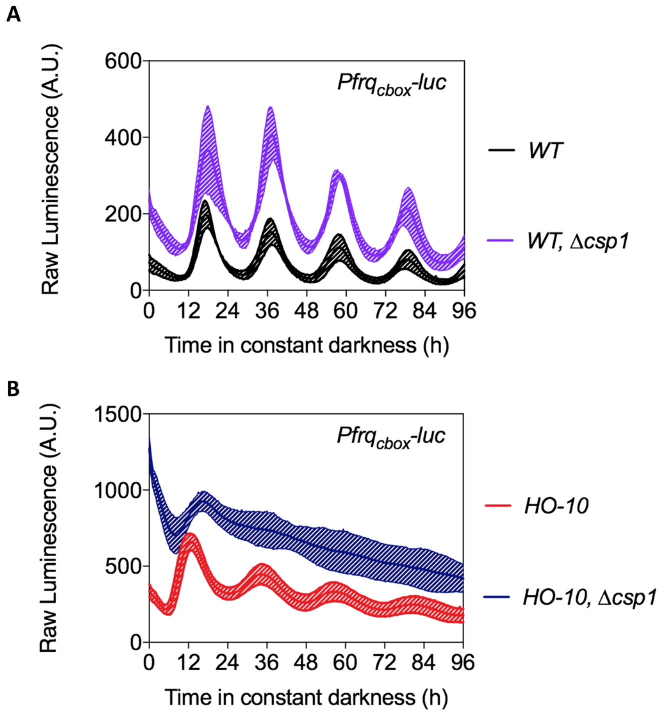

Figure EV3. Effect of the absence of *csp-1* in the WT and HO-10 oscillators.

(**A**,**B**) Evaluation of the WT (**A**) and HO-10 oscillator (**B**) in the absence of *csp-1*. Strains were entrained for 3 days under 12:12 LD cycles prior to monitoring in DD. Strains contain a *c-box-luc* reporter. In (**A**), the black traces represent the WT strain and purple traces the Δ*csp-1* in a WT clock background. In (**B**), the red traces represent the HO-10 and blue traces the Δ*csp-1* in a HO-10 background. In all cases, experiments were run three independent times, and a representative set is shown. Each luciferase trace corresponds to the average of three different wells ± SD.

 © The Author(s)

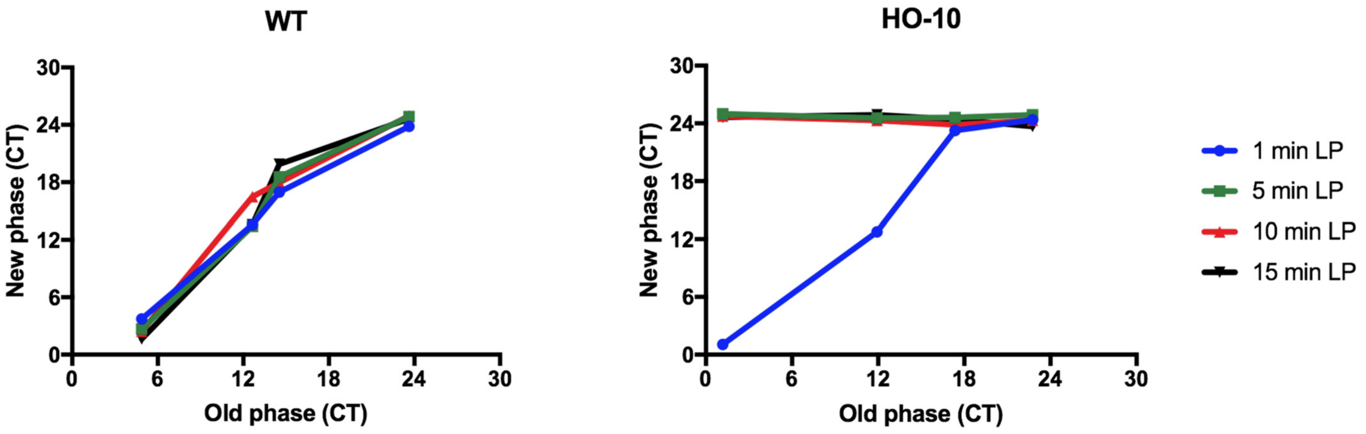

Figure EV4. HO-10 mainly exhibits type 0 PTCs.

The phase shift produced by discrete light pulses (LPs) of different duration was evaluated in a *WT* (left panel) and *HO-10* (right panel) clock. Strains were entrained as in Fig. 4G,H and after 48 h in DD and LP of 1, 5 10, and 15 min were administrated. While for all different tested LPs the WT clock exhibited a Type 1 PTC, the HO-10 depicted a type 0 PTC for LPs of 5, 10, and 15 min. Only for the shorter LP (of 1 min) a Type 1 PTC was obtained. The reported phase shifts were calculated by comparing the new phase after LP to the phase of the same strain without LP. $frq_{c\text{-}box+pLRE}$*-luc-PEST* was used as reporter. In all cases, experiments were run three independent times, and a representative set is shown. Each luciferase trace corresponds to the average of three different wells. Standard deviation is not plotted to help visualization of each independent PTC.

© The Author(s)

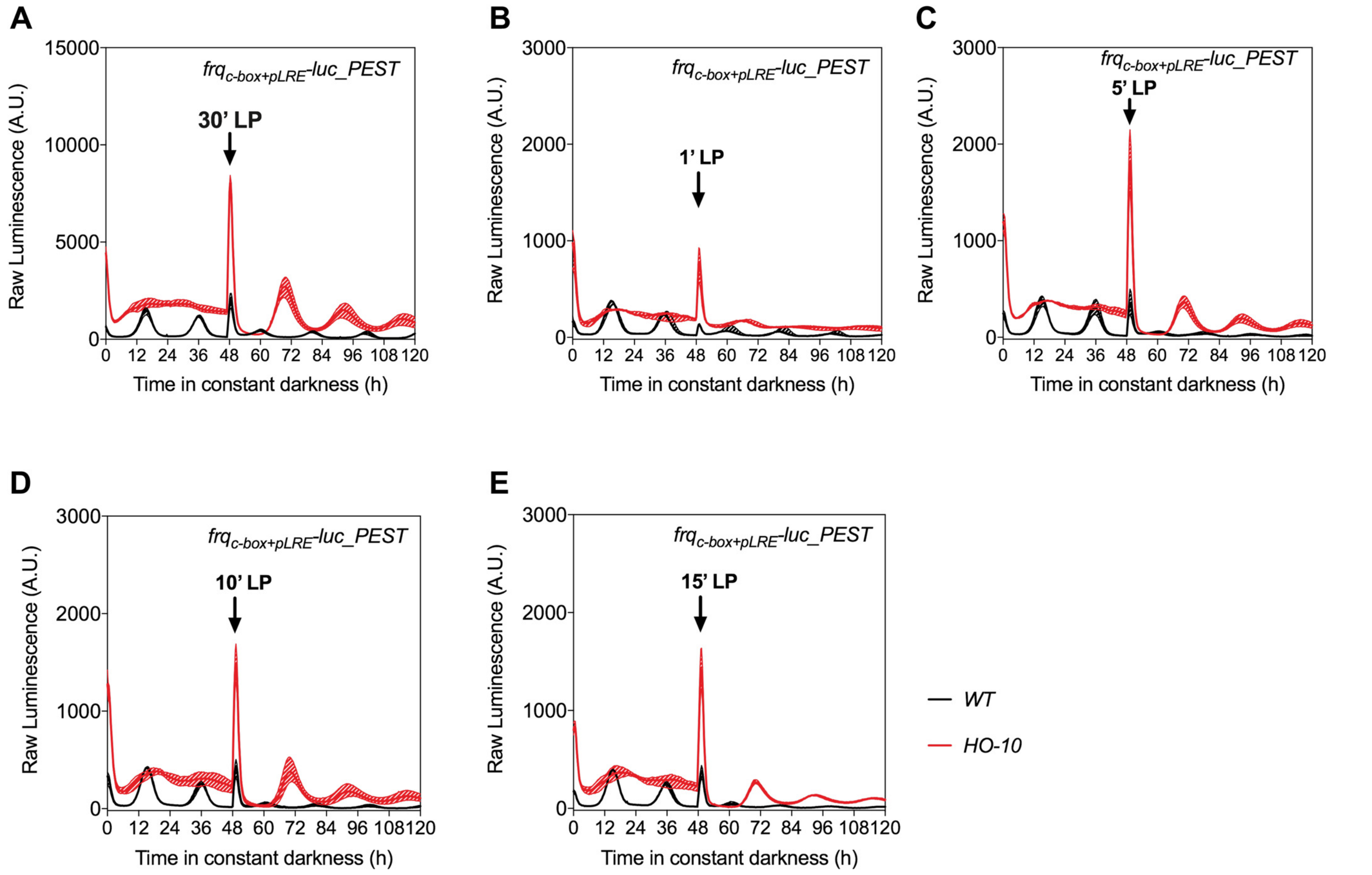

Figure EV5. A 5-min LP is sufficient to trigger oscillations in HO-10.

(**A–E**) After identifying that a 30 min LP could "jump start" the HO-10 (**A**), we evaluated the effect of different duration LPs in *WT* (black) and *HO-10* (red) strains. Strains were transfer from 24 h LL and start monitoring LUC activity in DD, after 48 h a LP of 1 (**B**), 5 (**C**), 10 (**D**), 15 **(E)** min was administrated. *frq*$_{c\text{-}box+pLRE}$*-luc-PEST* was used as a reporter. In all cases, experiments were run three independent times, and a representative set is shown. Each luciferase trace corresponds to the average of three different wells ± SD.

© The Author(s)
